# Supplementary material for: Integration of Inkjet Printed Graphene as a Hole Transport Layer in Organic Solar Cells
Source: Micromachines (Basel). 2023 Sep 28;14(10):1858. doi: 10.3390/mi14101858 (PMC10608915; doi:10.3390/mi14101858)
Supplement: Supplementary file 1 [file micromachines-14-01858-s001.zip › micromachines-2590850-supplementary.pdf]

SUPPORTING INFORMATION

Integration of Inkjet Printed Graphene as Hole Transport Layer in Organic Solar Cells

Julia Kastner[*,a], *Flavia Tomarchio*[b], *Nicolas Decorde*[b], *Matthias Kehrer*[c], *Günter Hesser*[c], *Anita Fuchsbauer*[a,d]

[a] Functional Surfaces and Nanostructures, Profactor GmbH, 4407 Steyr-Gleink, Austria

[b] Cambridge Graphene Centre, University of Cambridge, CB3 0FA, UK

[c] Center of Surface- and Nanoanalytics, Johannes Kepler University, 4040 Linz, Austria

[d] Current affiliation: Hueck Folien GmbH, 4342 Baumgartenberg, Austria

Corresponding Author
*E-mail: julia.kastner@profactor.at

S1 EXPERIMENTAL:
S1.1, Preparation of Graphene Dispersion. 2
S1.2. Characterization of Graphene Dispersion 2
S1.3. Plasma treatment of P3HT:PCBM and Characterization 3
S1.4. Inkjet Printing Procedure. 3

S2 RESULTS AND FIGURES:
S2.1. Characterization of Graphene Flakes 6
S2.2. Fabrication of Solar Cells with Graphene Ink as Hole-Transport Layer 8

REFERENCES

S1.1. Preparation of Graphene Dispersion

720 mg of graphite flakes (Sigma-Aldrich) are dispersed in 60 mL of deionized water (DIW) with 540 mg of sodium deoxycholate (SDC), then placed in an ultrasonic bath for 9 h at 37 kHz.

Subsequently, the dispersion is ultracentrifuged exploiting sedimentation-based separation (SBS) using a TH-641 swinging bucket rotor in a Sorvall WX-100 ultracentrifuge at 10 000 rpm (~17 000 g) for 1 h. The supernatant collected is the graphene standard ink.

In order to achieve a higher concentration of the graphene ink, keeping constant the quality, we demonstrate a liquid processing techniques, based on **iterative centrifugation** steps: the supernatant collected from the previous centrifuge step at 10 000 rpm is subjected to further centrifugation at 40 000 rpm for 3 h (274 000 g). After this step, the supernatant is discarded being only water, while the sediment after adding the standard ink is further centrifuged at the same speed. The process is repeated 3 times. Afterwards the sediment obtained from the iterative centrifuge steps is re-dispersed using a Diagenode Bioruptur® ultrasonication device with built-in cooling system for 2 h at maximum power. 10 wt% of propylene glycol is used to increase the viscosity to ~2.5 mPa s at 30°C.

S1.2. Characterization of Graphene Dispersion

The concentration of the dispersions was determined by optical absorption spectroscopy (OAS). The dispersions are diluted to 10% to avoid scattering losses at higher concentrations, and the absorption spectrum is acquired in the range of 200−1300 nm with an Agilent Cary 7000 UMA spectrophotometer. The concentration of graphitic flakes is determined from the optical absorption coefficient at 660 nm, using A = αlc where A is the absorbance, l [m] is the light path length, c [g/L] is the concentration of dispersed graphitic material, and α [L/g m] is the absorption coefficient, with α ~1390 L/g m at 660 nm.54 The concentration of the graphene ink obtained is ~1 g/L.

Raman spectroscopy was used to estimate the quality of the material produced by LPE. The Raman spectra are acquired at 457, 514 and 633 nm using a Renishaw InVia spectrometer equipped with 100x objective (N.A. = 0.85). The power on the sample is kept below 1 mW to avoid any possible thermal damage. To perform the measurements, the dispersion is deposited by drop cast on a 1x1 cm Si/SiO2 substrate, oxide thickness 285 nm to ensure the optical contrast. Around 60 µL of the aqueous solution is deposited on the silicon on a hot plate at 80-100°C for 20 minutes, to assure the evaporation of water. The samples are then washed with a mixture of water and ethanol 50:50 in volume in order to wash most of the surfactant away that interferes with the Raman measurements. Around 20 spectra are collected for the statistical analysis at the three wavelengths.

S1.3. Plasma treatment of P3HT:PCBM and characterization

Before printing onto the photopolymer surface P3HT:PCBM, the substrate Glass/ITO/ZnO/P3HT:PCBM was exposed to Argon plasma in the “Nano” plasma asher from Diener electronic GmbH & Co.KG (Germany) with a chamber size of 240x240x420 mm^3 and equipped with a Panasonic Timer LT4H. Gas was regulated to 16 sccm Ar gas (Messer Argon 6.0) for the sample treatment under 0.3 mbar. The treated samples are kept in Ar atmosphere before printing.

The surface was characterized by Surface Free Energy (SFE), Contact angle (CA), Atomic Force Microscopy (AFM) and X-ray Photoelectron Spectroscopy (XPS).

S1.4. Inkjet Printing Procedure

Devices were prepared by inkjet printing in ambient atmosphere but in rooms with UV reduced light conditions to avoid the light enhanced degeneration of the active polymer layer when exposed to oxygen. The graphene ink printed on pre-fabricated ITO/ZnO/P3HT:PCBM substrates (with 27 mm^2 active area) with a Dimatix Printer DMP-2831 and drop volume of 10 pL. The piezo voltage of the print head was set to 40 V. After the printing process, the device was annealed at 120°C for 3 min and the stabilizing surfactant SDC removed by rinsing with a mixture of H_2O:Ethanol 1:1 solution for few seconds and then dried with nitrogen.

After plasma treatment, the graphene ink was printed on Glass/ITO/ZnO/P3HT:PCBM substrates (with 27 mm^2 active area) with the Dimatix Printer in two rectangular shapes (as shown in Figure S1).

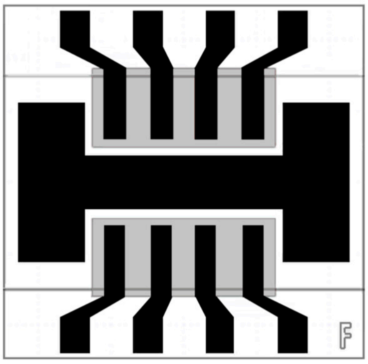

Figure S1. Scheme of the sample cell with graphene used as HTL. The graphene pattern (grey rectangles 13 x 27.6 mm) was printed on glass/ITO/ZnO/P3HT:PCBM (50 x 50 mm substrate). The solar cell is finished with evaporation of silver electrodes (black patterns). The two horizontal lines define laser lines cut through the ITO layer.

The waveform was adjusted to low viscosity water-based ink (compare Figure S2). The resulting average drop diameter on the plasma treated P3HT:PCBM substrate was 48.4 µm. The drop space defines the distance of the drops of ink printed. The best results were obtained by printing at room temperature with a drop space of 65 µm (printing resolution of 390.77 dpi). Nevertheless, it was not possible to avoid the coffee-ring-effect completely. A full coverage was achieved by shifting the printing origin prior to printing the next printing passes. From 10 to 20 printing passes were done to obtain a homogenous film of the graphene ink on the active polymer to avoid current leakage.

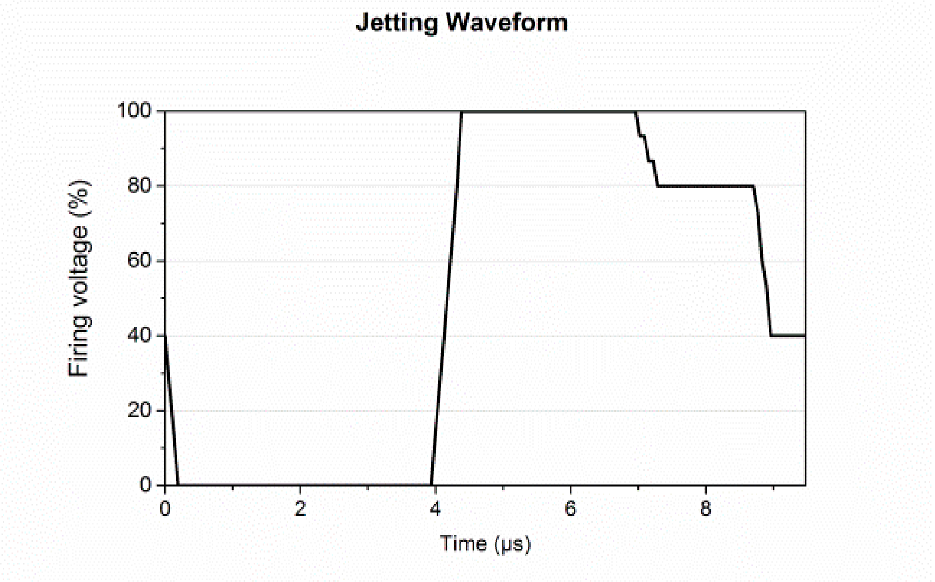

Segment	Level (%)	Slew Rate	Duration (μs)
**1**	0	2.00	3.904
**2**	100	2.00	3.008
**3**	80	0.60	1.728
**4**	40	1.33	0.832

Figure S2: A single pulse waveform was used for printing the graphene ink. The basic waveform for standard inks for the Dimatix printer DMP 2831 was adjusted to the low viscosity water-based graphene ink. Due to the low viscosity the overall pulse length was reduced to 9.472 µs. The jetting waveform consisted of four segments, with steep slew rates in the first two segments to ensure proper jetting of the dispersion.

RESULTS AND FIGURES:

S2.1. Characterization of graphene flakes

The Raman spectrum of graphene consists of a set of distinctive peaks; the G and 2D peak are the fingerprints for graphene. In presence of defects, more peaks arise, the D and D' peak. The G peak corresponds to the high frequency E_{2g} phonon at the Γ-point of the Brillouin zone of graphene.[55] The D peak is due to the breathing modes of six-atom rings and requires a defect for its activation.[56] It comes from transverse optical (TO) phonons around the Brillouin zone corner K.[55,56] It is active by double resonance (DR)[57,58] and is strongly dispersive with excitation energy due to a Kohn Anomaly (KA) at K[59,60]. Double resonance can also happen as an intravalley process, i.e. connecting two points belonging to the same cone around K (or K′). This gives the so-called D′ peak. The 2D peak is the D-peak overtone, and the 2D′ peak is the D′ overtone. Because the 2D and 2D′ peaks originate from a process where momentum conservation is satisfied by two phonons with opposite wave vectors, no defects are required for their activation, and are thus always present.[39,61,62] The 2D peak is a single Lorentzian in SLG, whereas it splits in several components as the number of layers' increases, reflecting the evolution of the electronic band structure.[61] In bulk graphite it consists of two components, roughly 1/4 and 1/2 the height of the G peak.[61] In disordered carbons, the position of the G peak, Pos(G), increases with decreasing of excitation wavelength (λL), resulting in a non-zero G peak dispersion, Disp(G) defined as the rate of change of Pos(G) with excitation wavelength. Disp(G) increases then with disorder.[63] Analogously to Disp(G), also the full width at half maximum of the G peak, FWHM(G), increases with disorder.[63] The analysis of the intensity ratio of the D to G peaks, I(D)/I(G), combined with that of FWHM(G) and Disp(G), allows one to discriminate between disorder localized at the edges and in the bulk. In the latter case, a higher I(D)/I(G) would correspond to higher FWHM(G) and Disp(G).

Figure S3a) plots a representative spectrum of the graphene produced by LPE. The average values for the Pos(G) (Figure S3b), and FWHM(G) (Figure S3c), are 1582.5 cm^{-1} and 23 cm^{-1} respectively. The line shape of the 2D band shows a of a single Lorentzian curve, and Pos(2D) (Figure S3d) is ~2698.5 cm^{-1}, and the FWHM(2D) (Figure S3e), is ~64 cm^{-1}. The I(2D)/I(G) (Figure S3g) ranges from 0.4 up to 1.04. These values suggests that the sample is a combination of mono layers and few layers graphene which are electronically decoupled that to a first approximation, behave as a collection of mono layers.[64] The Raman spectra show significant D and

D′ peaks intensity, with I(D)/I(G) ranging from 0.7 to 2.3 (Figure S3f). This is attributed to the edges of submicrometer graphene flakes, rather than to the presence of a large amount of structural defects within the flakes.[65] This observation is supported by the value of Disp(G) ~0.006 cm^{-1}/nm, much lower than the value expected for disordered carbon[55]. Moreover, the absence of correlation between I(D)/I(G) as a function of Disp(G) (Figure S3i) and FWHM(G) (Figure S3h) indicates that the D peak arises only from edges.

Figure S3: a) Representative Raman spectra of the graphene ink by LPE at 514.5 nm, Distribution of b) Pos(G), c)

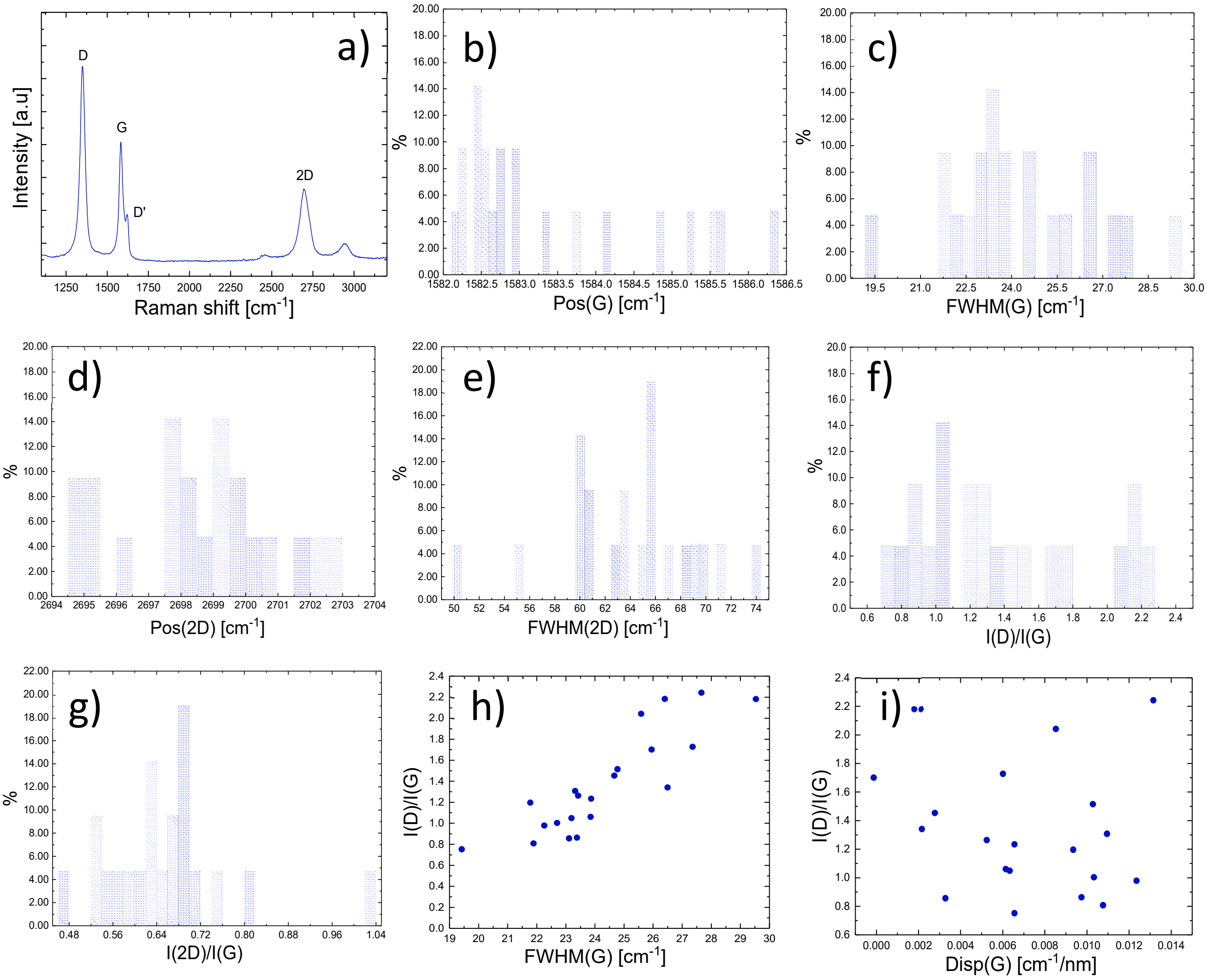

FWHM(G), d) Pos(2D), e) FWHM(2D), f) I(D)/I(G) and g) I(2D)/I(G). Distribution of I(D)/I(G) as a function of h) FWHM(G) and i) Disp(G).

S2.2. Fabrication of Solar Cells with Graphene Ink as Hole-Transport-Layer

The measured contact angles of water and calculated surface free energies (SFE) from water, ethylene glycol and diioiodomethane of untreated and plasma treated P3HT:PCBM are shown in Table S1.

Table S1. Results of SFE-measurements of the P3HT:PCBM layer.

**P3HT:PCBM substrate**	**Treatment-Details**	**Contact angle water (°)**	**Surface free energy (mN/m)**			**Wettability**
			Dispersive Part	Polar Part	**Total**	
**used as received**	---	**105.9**	25.0	0.1	**25.1**	Bad
**annealed**	130°C, 3 min	**99.4**	22.8	0.9	**23.7**	Bad
**Mild Ar plasma**	15 W, 6 sec	**79.1**	32.3	4.4	**36.7**	Good
**Ar plasma**	240 W, 1 min	**42.8**	34.7	21.1	**55.8**	Good

XPS measurements were done to study the treated surface more carefully (compare Figure S4a). The measurements revealed oxidation of the P3HT:PCBM surface after Ar plasma treatment: the O 1s peak is significantly increased from 3 atomic% for the untreated reference to 14 atomic% for the mild plasma treated and 21 atomic% for 240 W plasma treated P3HT:PCBM surface. Figure S4b indicates that with increasing plasma treatment power and time, more carbonyl and carboxyl species are formed matching the observation of the increasing of the polar part of SFE measurements. Carbonyl and carboxyl species cannot be seen in the spectrum of the untreated reference sample. More details on the fitting is shown in . As shown in Figure S4c, the C-S-C bound is heavily reduced, indicating most of the P3HT transformed by plasma into oxygen containing species. This is leading to a barrier in the solar cell with no proper charge transport.[66] Tountas *et al.* showed[67], that Ar Plasma is effecting especially the very out part of the polymer fitting to the result obtained that current is normally produced (no destroyed semiconducting system) however, the final charge transport is hindered.

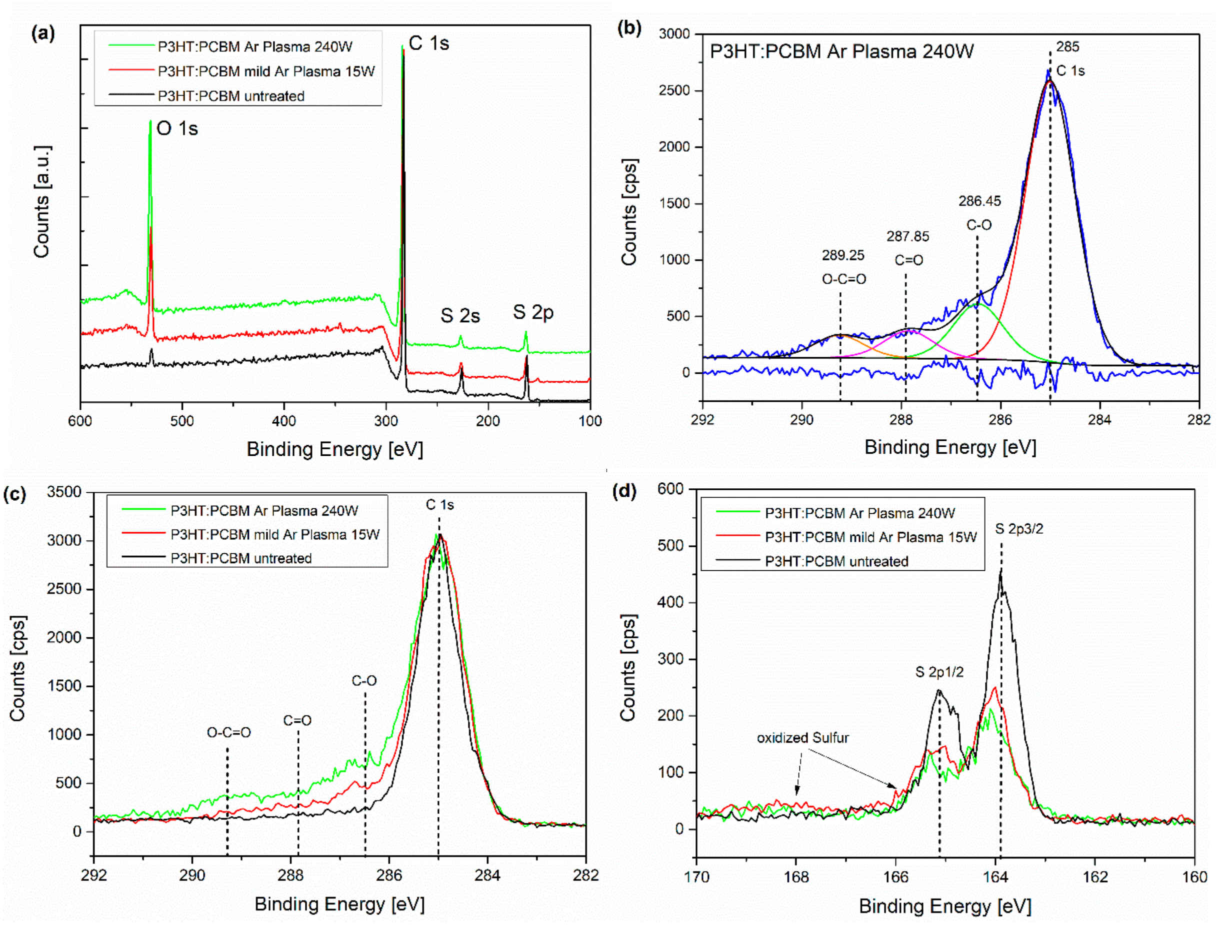

Figure S4: Results of the XPS measurements, showing the survey scan (a), fitting of C 1s peak (b), carbonyl and carboxyl peaks comparison of the C 1s regime (c) and of the S 2p peak (d).

Solar cells were made by doctor bladed standard PEDOT:PSS layer onto the plasma treated samples and a silver electrode evaporated to test the photovoltaic performance of the plasma treated devices. The *J-V* curves of the samples which were treated at 240 W Power for 1 min in Ar plasma showed a second diode behavior with very high threshold voltage needed (compare Figure S5) supporting the results of the XPS measurement. Current was produced - short circuit current (J_{sc}) was 7.22 mA/cm², but the opening behavior was shifted and charge carriers could only be extracted by applying high electric fields of more than 1 V. Consequently, no current could be collected from these treated solar cells. The samples treated with mild plasma conditions (15 W Power, 6 sec) showed no significant changes in the device performance although XPS measurements indicate beginning oxidation of the surface.

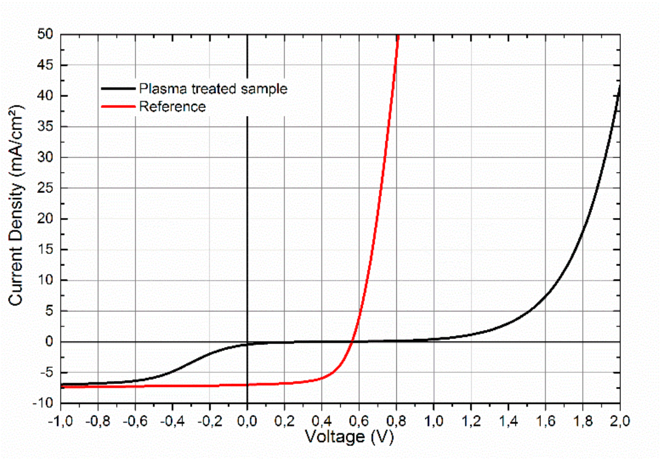

Figure S5: J-V curve for plasma treated (80% Power, 1 min) solar cells (black). The red line is the reference sample with no plasma treatment.

The results of the AFM measurements of graphene printed on the ITO/ZnO/P3HT:PCBM(mild plasma treated) stack is shown in Figure S6.

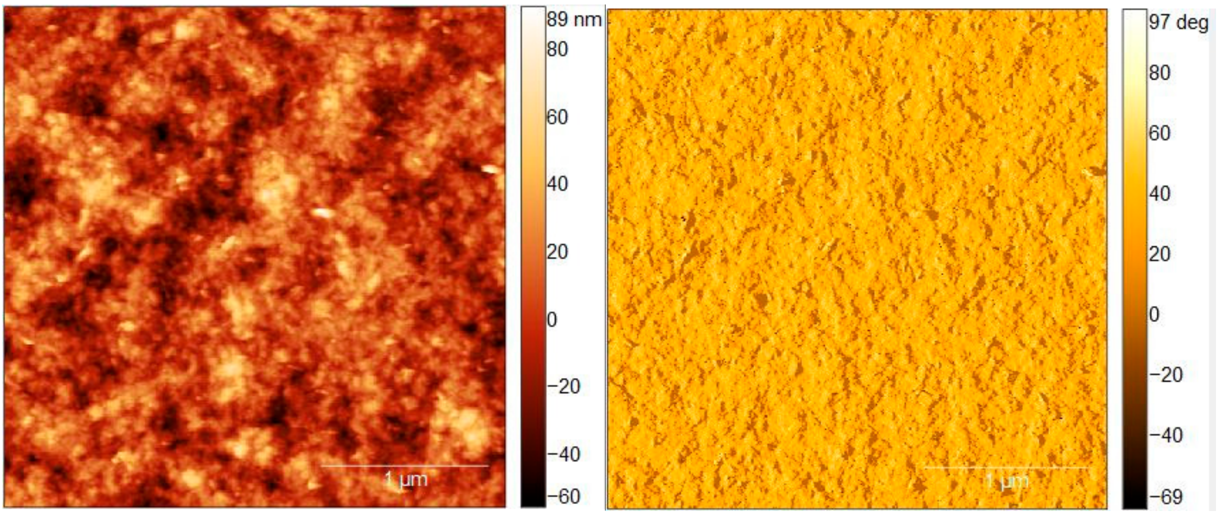

Figure S6: AFM of a printed graphene film on P3HT:PCBM (10 printing passes). Left: height profile, right: phase image.

Only few Ag signals can be detected in the TEM/EDX measurements of the fabricated solar cells with graphene as HTL (Figure S7).

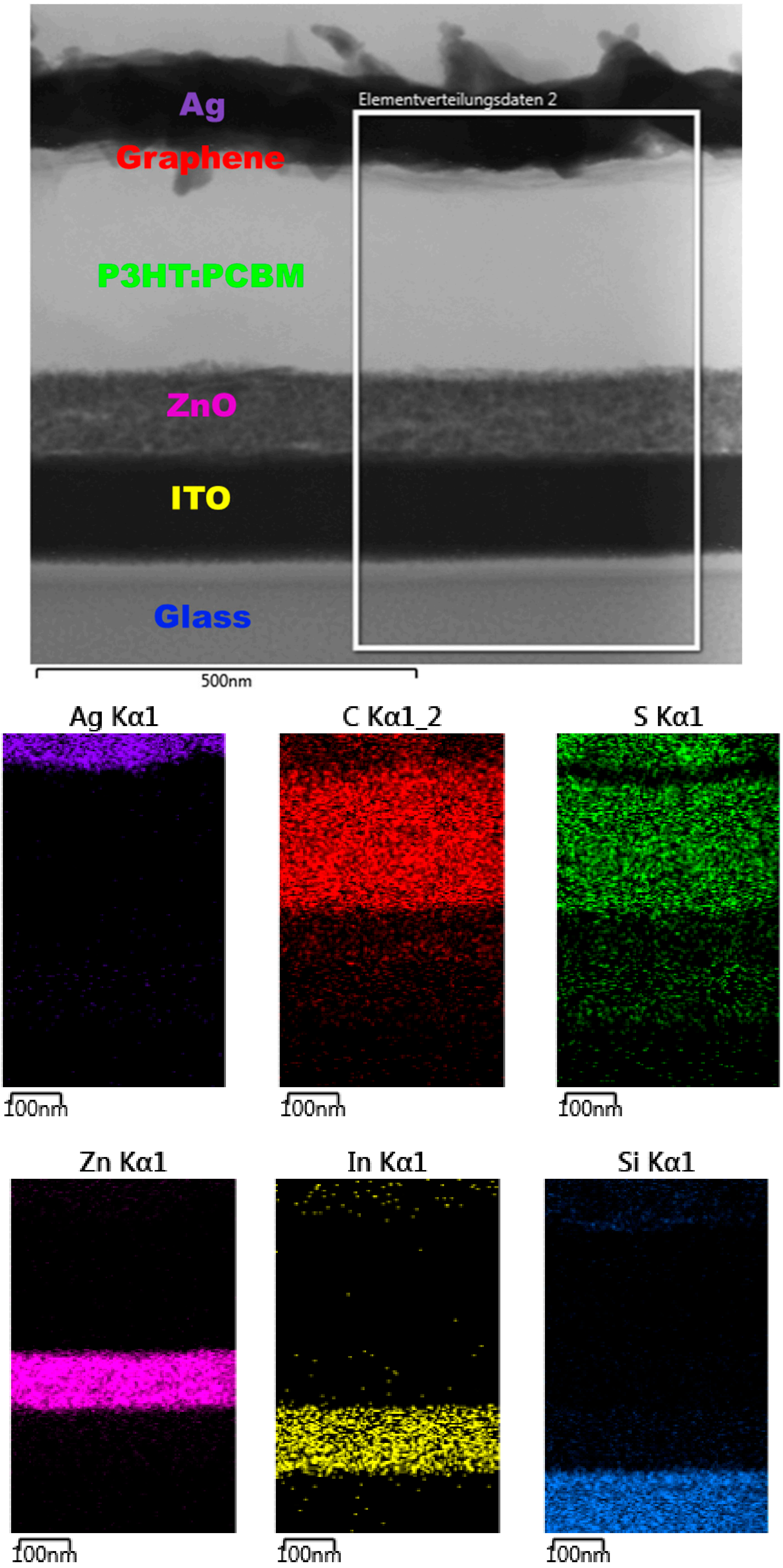

Figure S7: Cross-section via TEM/EDX showing the elemental dristribution in the different layers.

REFERENCES:

[1] F. Bonaccorso, A. Lombardo, T. Hasan, Z. Sun, L. Colombo, A.C. Ferrari, Production and processing of graphene and 2d crystals, Mater. Today. 15 (2012) 564–589.

[2] A.C. Ferrari, J. Robertson, Interpretation of Raman spectra of disordered and amorphous carbon, Phys. Rev. B. 61 (2000) 14095.

[3] C. Thomsen, S. Reich, Double resonant raman scattering in graphite, Phys. Rev. Lett. 85 (2000) 5214–5217. doi:10.1103/PhysRevLett.85.5214.

[4] A.V. Baranov, A.N. Bekhterev, Y.S. Bobovich, V.I. Petrov, Interpretation of certain characteristics in Raman spectra of graphite and glassy carbon, Opt. Spectrosc. 62 (1987) 612–616.

[5] I. Pócsik, M. Hundhausen, M. Koós, L. Ley, Origin of the D peak in the Raman spectrum of microcrystalline graphite, J. Non-Cryst. Solids. 227–230 (1998) 1083–1086. doi:10.1016/S0022-3093(98)00349-4.

[6] S. Piscanec, M. Lazzeri, F. Mauri, A.C. Ferrari, J. Robertson, Kohn Anomalies and Electron-Phonon Interactions in Graphite, Phys. Rev. Lett. 93 (2004) 185503. doi:10.1103/PhysRevLett.93.185503.

[7] A.C. Ferrari, J.C. Meyer, V. Scardaci, C. Casiraghi, M. Lazzeri, F. Mauri, S. Piscanec, D. Jiang, K.S. Novoselov, S. Roth, A.K. Geim, Raman Spectrum of Graphene and Graphene Layers, Phys. Rev. Lett. 97 (2006) 187401. doi:10.1103/PhysRevLett.97.187401.

[8] D.M. Basko, S. Piscanec, A.C. Ferrari, Electron-electron interactions and doping dependence of the two-phonon Raman intensity in graphene, Phys. Rev. B. 80 (2009) 165413. doi:10.1103/PhysRevB.80.165413.

[9] M. Bruna, A.K. Ott, M. Ijäs, D. Yoon, U. Sassi, A.C. Ferrari, Doping Dependence of the Raman Spectrum of Defected Graphene, ACS Nano. 8 (2014) 7432–7441. doi:10.1021/nn502676g.

[10] F. Torrisi, T. Hasan, W. Wu, Z. Sun, A. Lombardo, T.S. Kulmala, G.-W. Hsieh, S. Jung, F. Bonaccorso, P.J. Paul, D. Chu, A.C. Ferrari, Inkjet-Printed Graphene Electronics, ACS Nano. 6 (2012) 2992–3006. doi:10.1021/nn2044609.

[11] A.C. Ferrari, S.E. Rodil, J. Robertson, Interpretation of infrared and Raman spectra of amorphous carbon nitrides, Phys. Rev. B. 67 (2003) 155306. doi:10.1103/PhysRevB.67.155306.

[12] A.C. Ferrari, Raman spectroscopy of graphene and graphite: Disorder, electron–phonon coupling, doping and nonadiabatic effects, Solid State Commun. 143 (2007) 47–57. doi:10.1016/j.ssc.2007.03.052.

[13] C. Casiraghi, A. Hartschuh, H. Qian, S. Piscanec, C. Georgi, A. Fasoli, K.S. Novoselov, D.M. Basko, A.C. Ferrari, Raman Spectroscopy of Graphene Edges, Nano Lett. 9 (2009) 1433–1441. doi:10.1021/nl8032697.
